# *Aspidistra crassifila* (Asparagaceae), a new species from Guangxi, China

**DOI:** 10.1186/1999-3110-54-43

**Published:** 2013-10-07

**Authors:** Chun-Rui Lin, Yan Liu, Dong-Xin Nong, Yoshiko Kono, Ching-I Peng

**Affiliations:** 1 Guangxi Institute of Botany, Guangxi Zhuangzu Autonomous Region and the Chinese Academy of Sciences, Guilin, 541006 P.R. China; 2 Guangxi Botanical Garden of Medicinal Plants, Nanning, 530023 P.R. China; 3 Herbarium (HAST), Biodiversity Research Center, Academia Sinica, Nangang, Taipei, 115 Taiwan

**Keywords:** *Aspidistra crassifila*, *Aspidistra subrotata*, Asparagaceae, New species, Chromosome number, Karyotype, Guangxi, China

## Abstract

**Background:**

*Aspidistra crassifila* Yan Liu & C.-I Peng, a new species of the Asparagaceae from Guangxi Zhuang Autonomous Region, China, is described and illustrated.

**Results:**

The new species is similar to *A. subrotata* Y. Wan & C. C. Huang in the perianth lobes triangular-lanceolate and horizontally spreading, but differs by the perianth campanulate, lobes with appendages at base, stamens 6–8 mm long, filaments enlarged, anthers adnate to perianth tube, connectives extended and upcurved. The chromosome number of the new species was determined to be 2*n* = 38, and the karyotype was formulated as 2*n* = 22m^2SC^+4sm+12st.

**Conclusion:**

A careful study of the literature, herbarium specimens and living plants, both in the wild and in cultivation in the experimental greenhouse, support the recognition of the new species *Aspidistra crassifila*, which is described herein. *Aspidistra crassifila* is currently known only from Shiwandashan Mountains, which lie in southern Guangxi. A line drawing, color plates and a distribution map are given for the new species to aid in identification.

**Electronic supplementary material:**

The online version of this article (doi:10.1186/1999-3110-54-43) contains supplementary material, which is available to authorized users.

## Background

The genus *Aspidistra* Ker-Gawler (Asparagaceae) was established in 1822 and is comprised of ca. 100 species, more than 60 of which occur in China (Lang et al., 1999; Li, 2004; Tillich, 2005,2008; Hou et al., 2009; Lin et al., 2009,2010; Liu et al., 2011). In March 2005, during a field trip to Shiwandashan Mountains in southern Guangxi Zhuang Autonomous Region, China, the second author (Yan Liu) collected and brought back a sterile plant of *Aspidistra* for cultivation, which flowered in Guilin Botanical Garden next spring. We went on another trip to the same locality in Shiwandashan Mountains in January 2007 and were able to collect fruiting materials of this species. Compared with other species of *Aspidistra*, it was recognized as an undescribed species that differs from congeners in its peculiar adnate stamens with extended and upcurved connectives.

## Methods

### Chromosome preparations

Somatic chromosomes were examined for plants collected from the type collection (*Yan Liu L1380*). Root tips were pretreated in 2 mM 8-hydroxyquinoline at 15-18°C for about 8 h, then fixed overnight in a 3:1 ethanol-acetic acid solution below 4°C. The chromosomes were stained with 2% acetic orcein in 1mol/L hydrochloric acid and observed. Classification of chromosome morphology is based on the position of the centromere, following Levan et al. (1964).

## Results and discussion

### Taxonomic treatment

**Figure 1:**
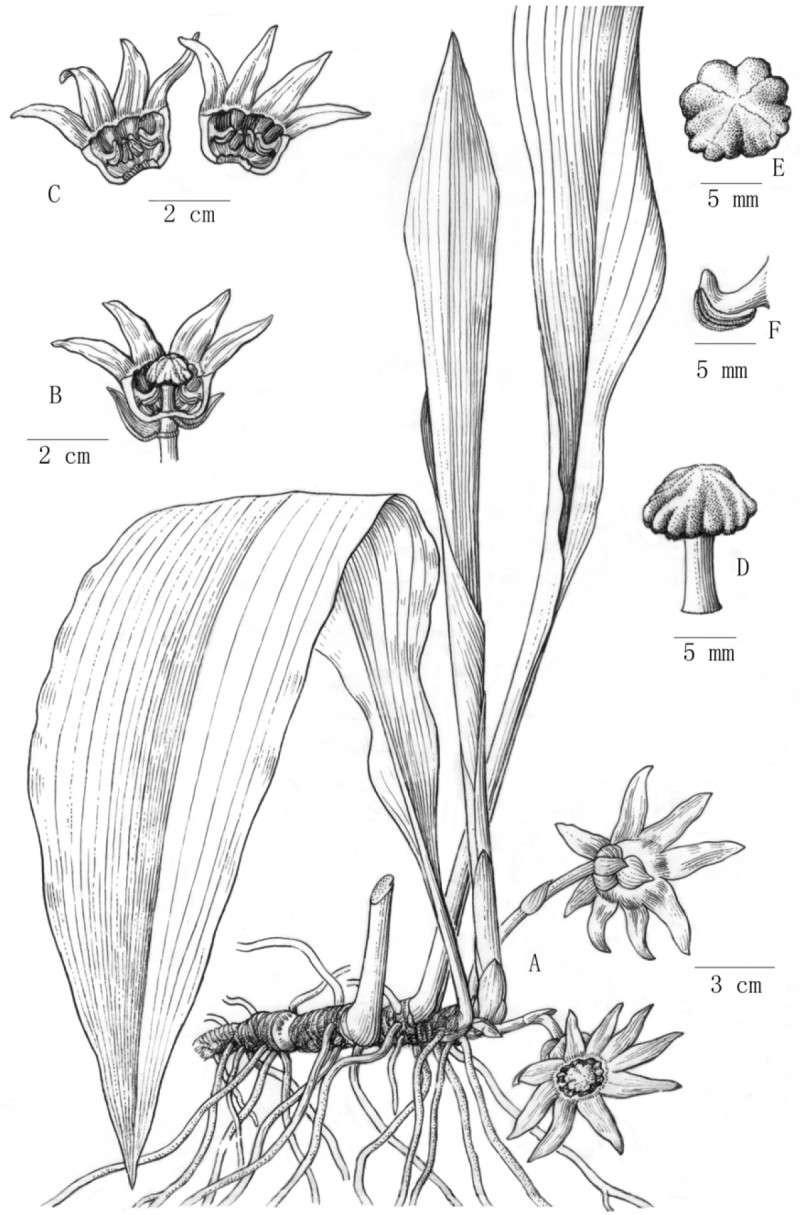
***Aspidistra crassifila*** **Yan Liu & C.-I Peng. A**, Flowering plant; **B**, Flower with half of perianth removed showing stamens and pistil; **C**, Perianth, dissected to show stamens; **D**, Pistil; **E**, Stigma, adaxial view; **F**, Stamen. (Drawn by Shun-Qing He from the holotype).

**Figure 2:**
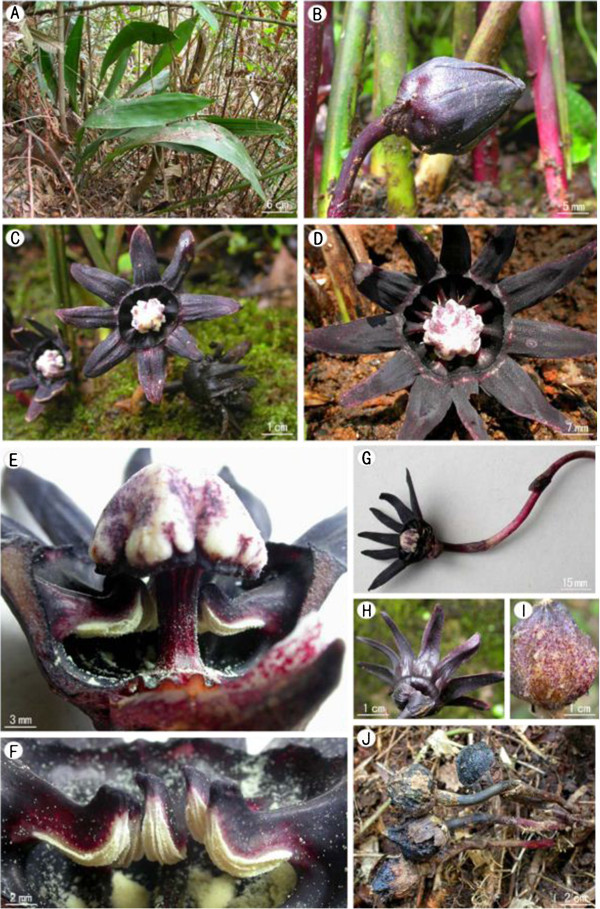
***Aspidistra crassifila*** **Yan Liu & C.-I Peng. A**, Habit; **B**, Bud; **C**, **D**, Flowers; **E**, Flower with half of perianth removed showing stamens and pistil; **F**, Stamen; **G**, Flower with peduncle; **H**, Flower, abaxial view; **I**, **J**, Fruit.

**Aspidistra crassifila** Yan Liu & C.-I Peng, sp. nov.—TYPE: CHINA. Guangxi Zhuang Autonomous Region, Fangcheng (City), Shiwandashan Mountains, alt. 980 m, 10 March 2005. Specimens pressed from plants introduced to Guilin Botanical Garden, Guilin City, Yanshan Township on 11 May 2006, *Yan Liu L1380* (holotype: IBK; isotype: HAST) 粗丝蜘蛛抱蛋 Figures 1, 2.

Species nova A. subrotatae Y. Wan & C. C. Huang affinis, sed differt perianthio campanulato (vs. subrotato), lobis basi appendiculatis (vs. non appendiculatis, margine reflexis), filamentis dilatatis (vs. non dilatatis), antheris adnatis (vs. versatilibus), connectivis supra antheras productis (vs. non productis).

#### Description

Herbs perennial, evergreen, rhizomatous. Rhizome creeping, subterete, 8–13 mm thick, covered with scales, nodes dense. Vaginal leaves 4–5, 1–14 cm long, purple-red, enveloping base of petiole, fibrous when withered. Leaves solitary, 2–5 cm apart; petiole stiff , upright, 10–40 cm long, 3–5 mm thick, adaxially sulcate; leaf blade oblong-oblanceolate, 30–60 cm long, 6–12 cm wide, base cuneate, gradually narrowed into petiole, inequilateral, apex acuminate, margin entire. Peduncle purplish red to purplish black, 2.5-6 cm long, with 4–5 bracts, bracts gradually wider from base to top of peduncle; the uppermost bract broadly ovate, purplish red or purplish black, ca. 10 mm long, ca. 12 mm wide, apex subobtuse. Flowers solitary; perianth purplish black, fleshy, campanulate, 4–6 cm in diam., 8–12 lobed apically; lobes triangular-lanceolate, 15–25 mm long and 5–10 mm wide at base, apex gradually acuminate, horizontally spreading, with appendages at base, tube 8–12 mm long, distal opening 15–20 mm diam.; stamens as many as and opposite to lobes, 6–8 mm long, inserted in the middle of perianth tube, positioned lower than stigma, filaments purplish black, enlarged, 3–4 mm wide at side view, their upper surfaces visible from above (between adaxial surface of perianth tube and margin of stigma), anthers adnate to perianth, pale yellow, oblong, 5–6 mm long, ca. 2 mm wide, connectives extended and upcurved; pistil mushroom-shaped, 2 cm long, style cylindrical, ca. 10 mm long, ca. 4 mm across, purplish red, ovary inconspicuous, stigma enlarged, ca. 10 mm high, 12–15 mm across, upper surface white with purple spots, smooth, lower surface purplish black, irregularly concaved and undulate at margin. Berry subglobose, ca. 3.5 cm across, tuberculate.

#### Additional specimens examined

CHINA. Guangxi Zhuang Autonomous Region, Fangcheng (City), Shiwandashan Mountains, alt. 980 m, 10 March 2005, *Yan Liu L1156* (IBK); same locality, 17 January 2007, *Yan Liu L1425* (IBK).

#### Chromosome cytology

**Figure 3:**
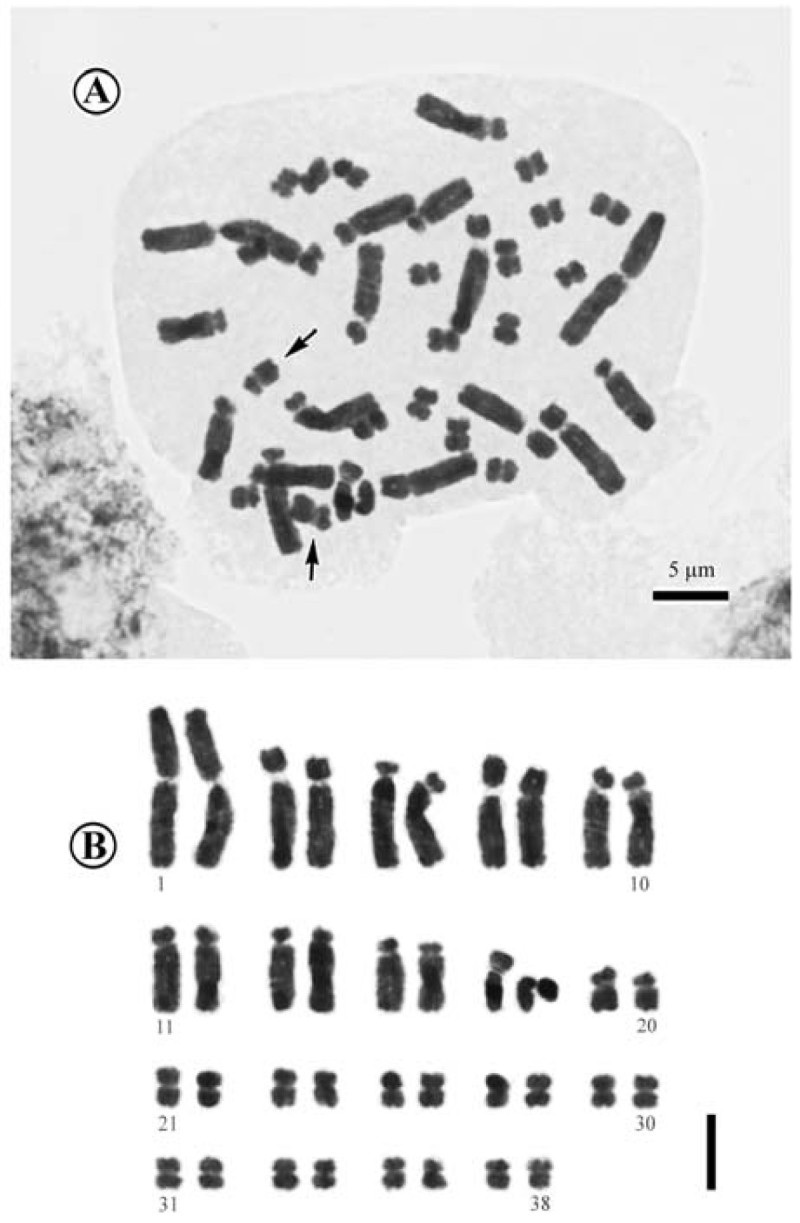
**Somatic chromosomes at metaphase of** ***Aspidistra crassifila*** **(2** ***n*** **= 38, from** ***Yan Liu L1380*** **, HAST). A**, Microphotograph. Arrows indicate median chromosomes with secondary constrictions; **B**, Somatic chromosomes serially arranged by their length and the position of centromeres. Scale bar = 5 μm.

The chromosome number of *Aspidistra crassifila* was determined to be 2*n* = 38 (Figure 3), showing a trimodal variation in chromosome length at mitotic metaphase. Among the 38 chromosomes, the first two were much longer (ca 10.5-10.6 μm) than the rest; the next 16 gradually varied, ca 3.9-7.5 μm; the remaining 20 chromosomes also gradually varied, ca. 1.9-2.8 μm long. Regardless of the chromosome length, 22 (Nos. 1, 2 and 19–38 in Figure 3B), 4 (Nos. 7, 8 and 17, 18 in Figure 3B), 12 (Nos. 3–6 and 9–16 in Figure 3B) had centromere at the median (m), submedian (sm), and subterminal (st) positions, respectively. Secondary constrictions (SC) were observed at the proximal regions of the short arms in two submedian chromosomes (arrows in Figure 3A; Nos. 19 and 20 in Figure 3B). Thus, the karyotype formula of *Aspidistra crassifila* is 2*n* = 38 = 22m^2SC^+4sm+12st.

Previously, detailed cytological data were known for 42 species in the genus *Aspidistra* (Bogner and Arnautov, 2004; Li, 2004; Yamashita and Tamura, 2004; Qiao et al., 2008; Hou et al., 2009, Lin et al., 2010; Liu et al., 2011). *Aspidistra crassifila* shared a number of cytological features, namely basic chromosome numbers of *x* = 19, trimodal chromosome complement, the first pair of longer median chromosomes and 10th median SC-chromosomes, in common with those of the *Aspidistra* species previously reported (Lin et al., 2010).

#### Ecology

On shaded bamboo slopes in seasonal rain forests (monsoon forests).

#### Distribution

**Figure 4:**
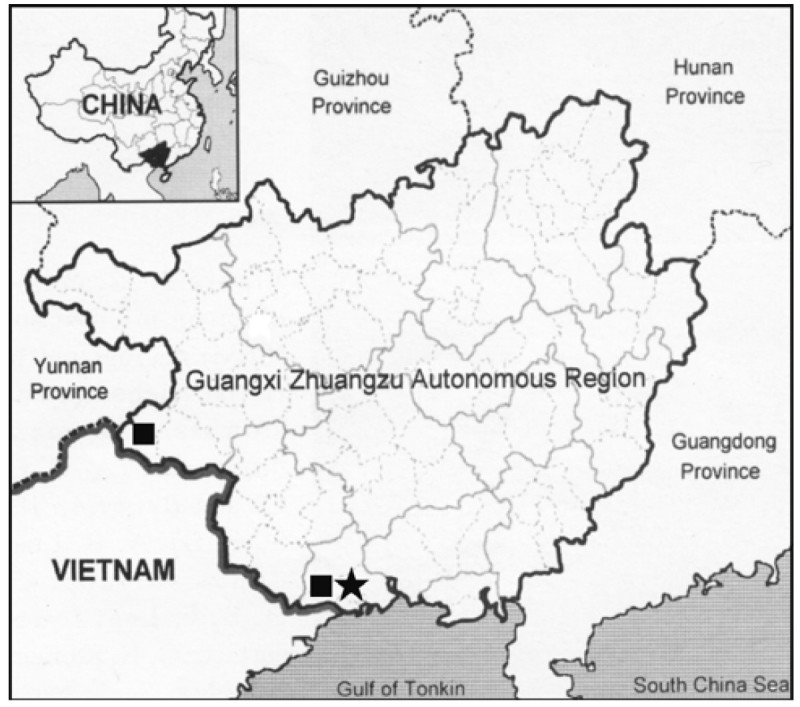
**Distribution of** ***Aspidistra crassifila*** **Yan Liu & C.-I Peng (★) and** ***A. subrotata*** **Y. Wan & C. C. Huang (■) in Guangxi Zhuangzu Autonomous Region, China.**

Currently known only from Shiwandashan Mountains, Fangcheng (City), in southern Guangxi Zhuang Autonomous Region, China (Figure 4).

#### Phenology

Flowering from March to May; fruits maturing in May next year.

#### Etymology

The specific epithet '*crassifila*' is derived from its enlarged filaments.

#### Notes

**Figure 5:**
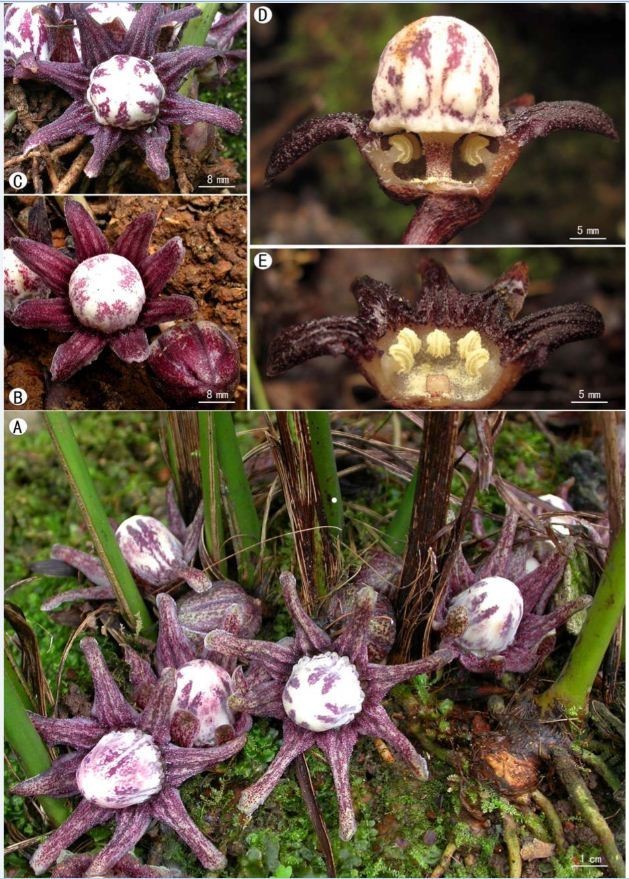
***Aspidistra subrotata*** **Y. Wan & C. C. Huang. A**, Habit; **B**, **C**, Flowers; **D**, **E**, Flower, dissected to show stamens and pistil.

*Aspidistra crassifila* (Figures 1, 2) resembles *A. subrotata* Y. Wan & C. C. Huang (Figure 5; Wan and Huang, 1987) in the perianth lobes triangular-lanceolate, horizontally spreading, but differs in its perianth campanulate (vs. subrotate), lobes with appendages at base (vs. without appendages, margin reflexed), filaments enlarged (vs. non enlarged), anthers adnate (vs. versatile) to parianth. Connectives extended and upcurved (vs. not extended). Also the purplish black perianth color is rather rare in *Aspidistra*, known only from *A. renatae* C. Bräuchler (Bräuchler and Ngoc, 2005), *A. nikolai* L.V. Averyanov & H.-J. Tillich (Tillich and Averyanov, 2008), *A. atroviolacea* H.-J. Tillich (Tillich, 2008), and *A. pileata* D. Fang & L. Y. Yu (Fang and Yu, 2002).

## Conclusion

A careful study of the literature, herbarium specimens and living plants, both in the wild and in cultivation in the experimental greenhouse, support the recognition of the new species *Aspidistra crassifila*, which is described herein. *Aspidistra crassifila* is currently known only from Shiwandashan Mountains, which lie in southern Guangxi.

## Authors’ original submitted files for images

Below are the links to the authors’ original submitted files for images. Authors’ original file for figure 1 Authors’ original file for figure 2 Authors’ original file for figure 3 Authors’ original file for figure 4 Authors’ original file for figure 5
