# Supplementary figures and images for: Aspidistra crassifila (Asparagaceae), a new species from Guangxi, China
Source: Bot Stud. 2013 Oct 7;54:43. doi: 10.1186/1999-3110-54-43 (PMC5430311; doi:10.1186/1999-3110-54-43)

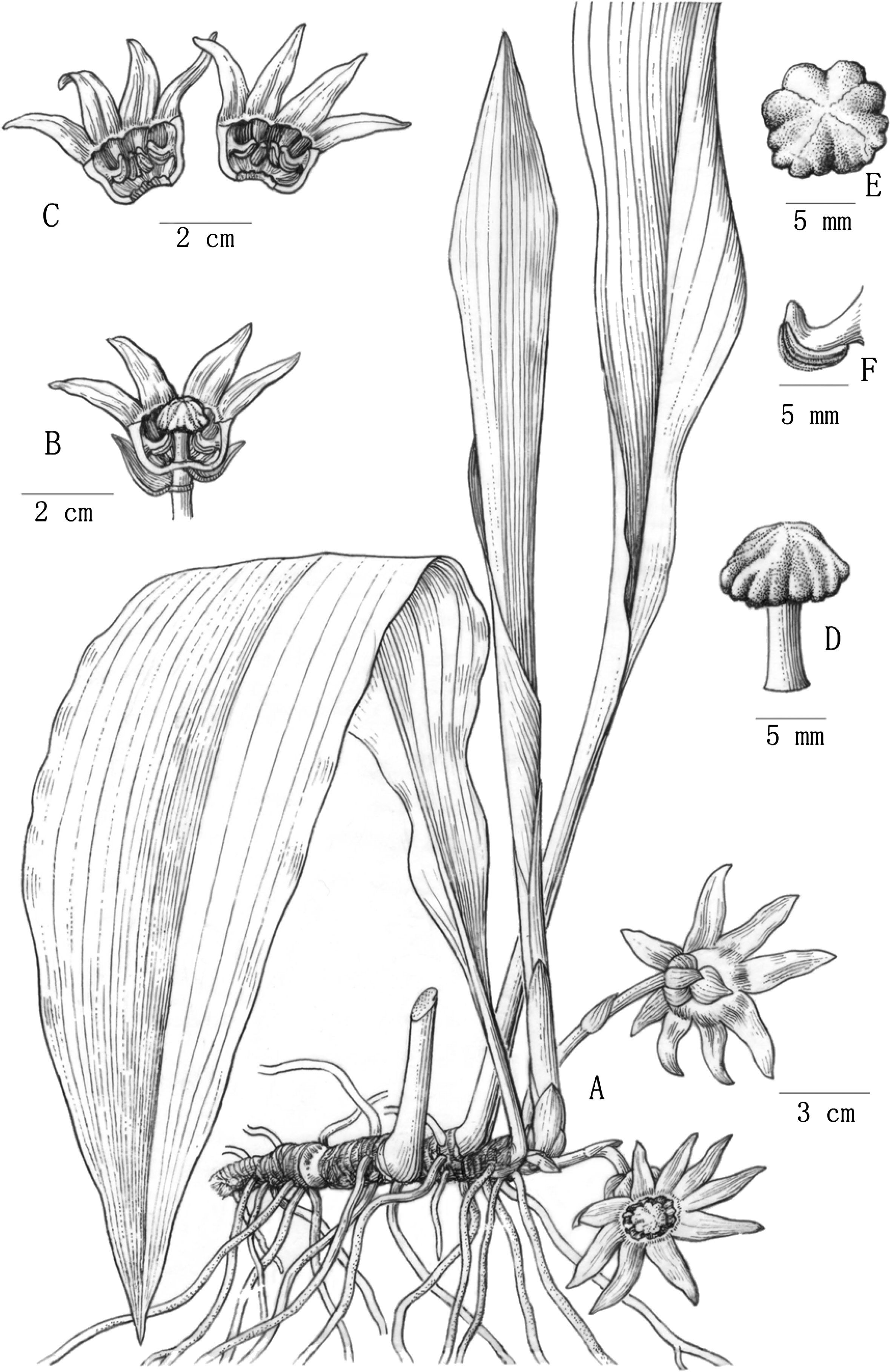

Supplement: Supplementary file 1 — Authors’ original file for figure 1 [file 40529_2013_95_MOESM1_ESM.tif]

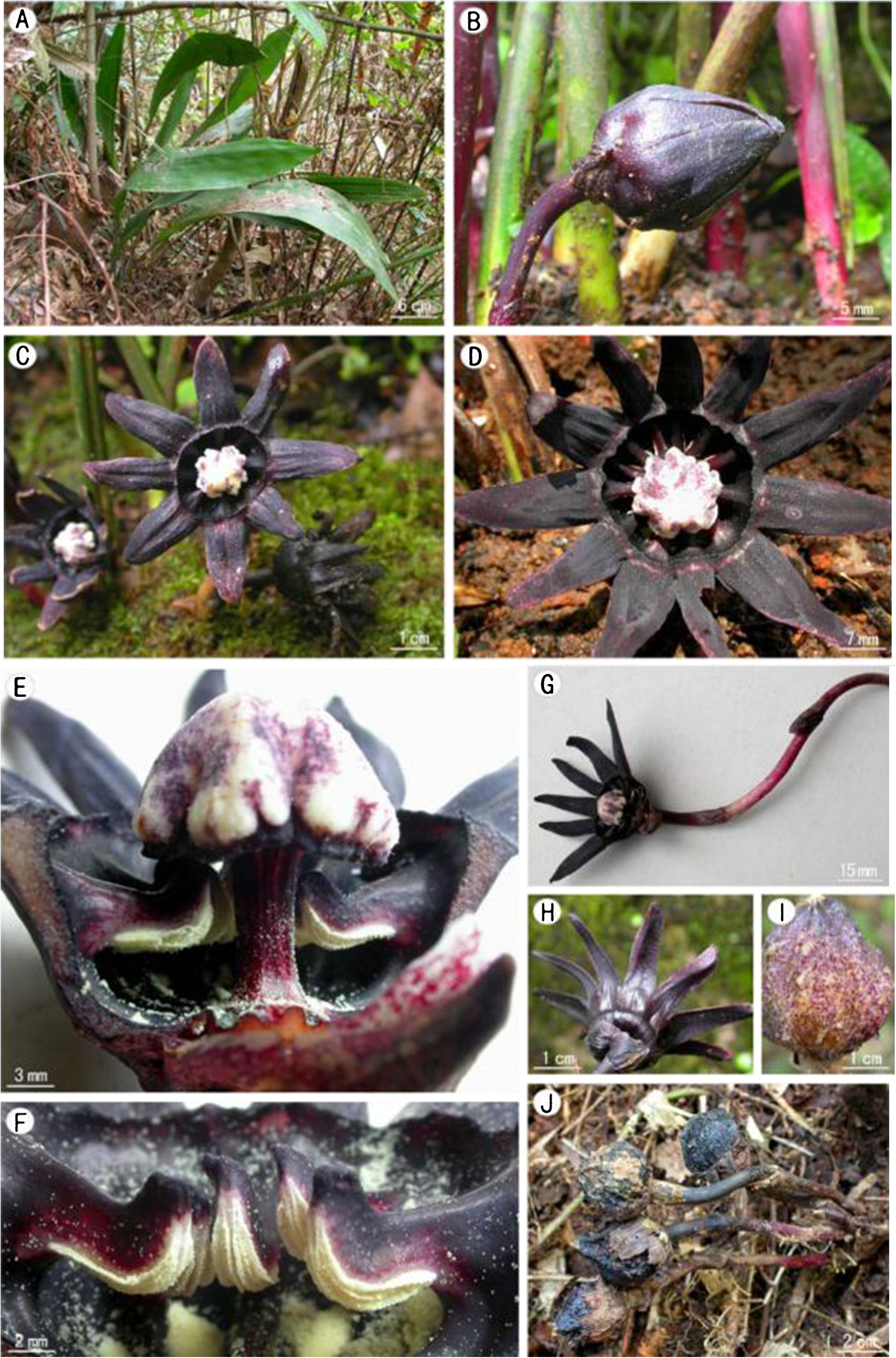

Supplement: Supplementary file 2 — Authors’ original file for figure 2 [file 40529_2013_95_MOESM2_ESM.tif]

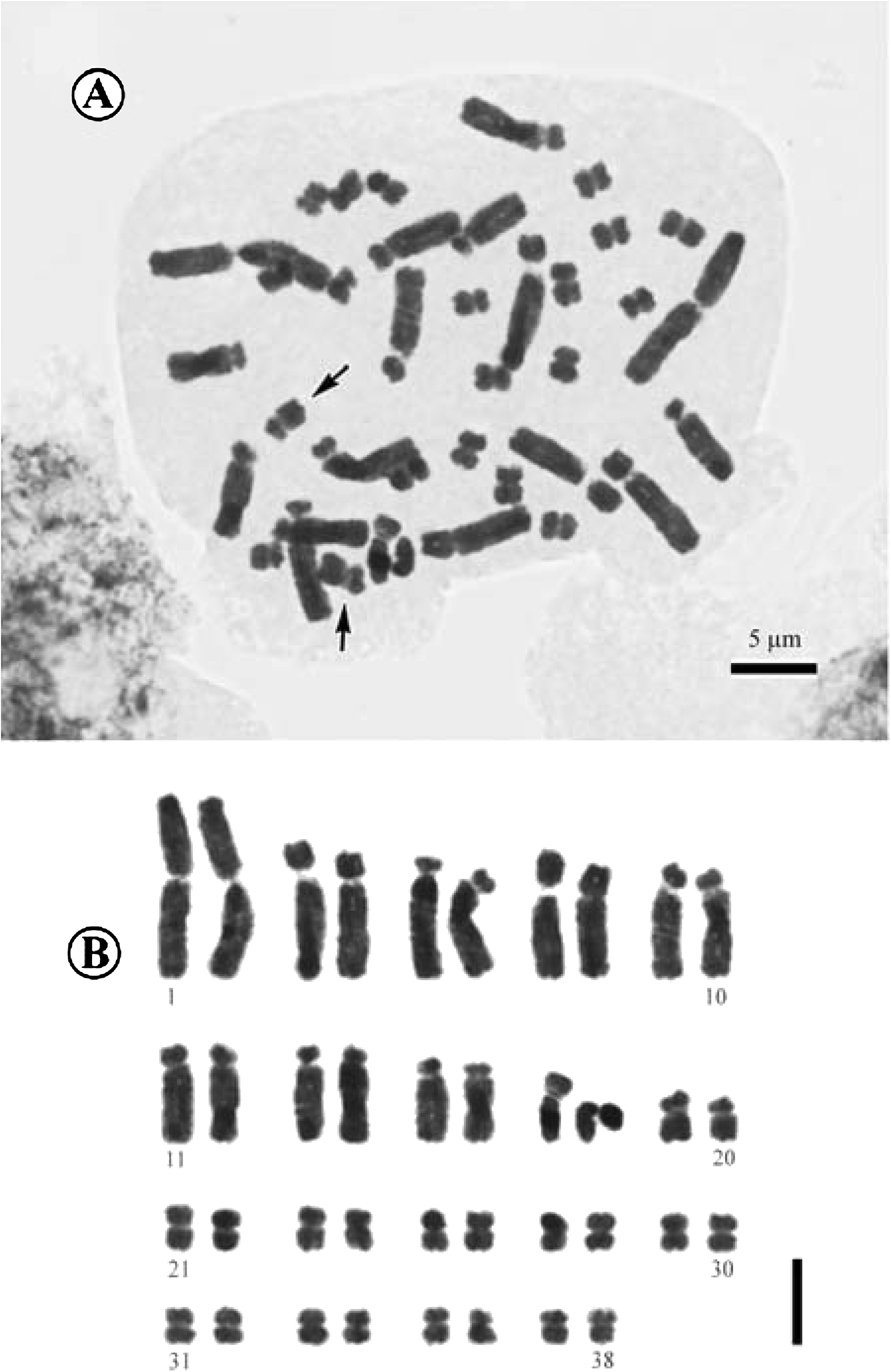

Supplement: Supplementary file 3 — Authors’ original file for figure 3 [file 40529_2013_95_MOESM3_ESM.tif]

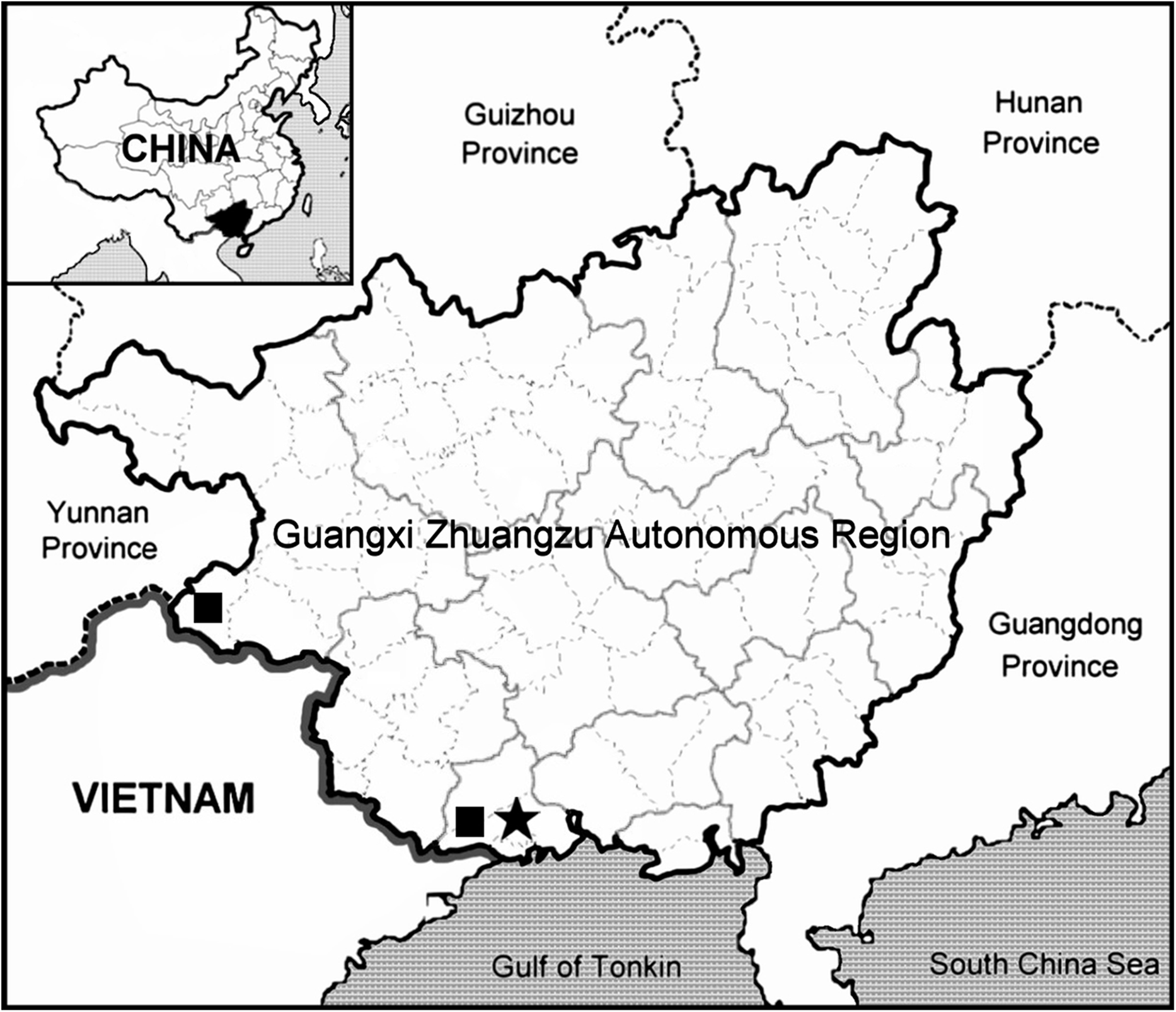

Supplement: Supplementary file 4 — Authors’ original file for figure 4 [file 40529_2013_95_MOESM4_ESM.tiff]

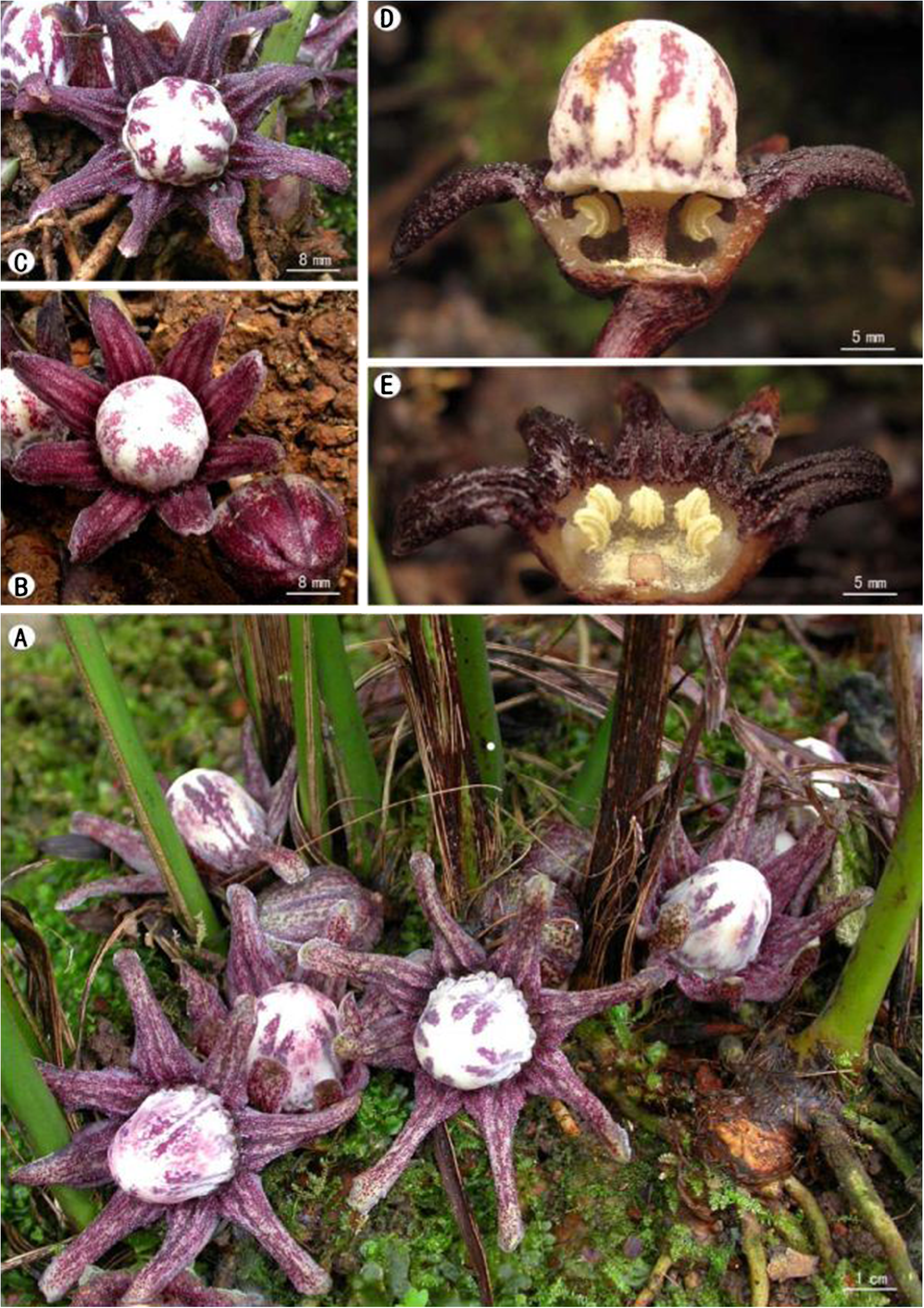

Supplement: Supplementary file 5 — Authors’ original file for figure 5 [file 40529_2013_95_MOESM5_ESM.tif]
